# Effect of Solid Waste-Petroleum Coke Residue on the Hydration Reaction and Property of Concrete

**DOI:** 10.3390/ma12081216

**Published:** 2019-04-13

**Authors:** Liang Wang, Hongzhu Quan, Qiuyi Li

**Affiliations:** College of Civil Engineering & Architecture, Qingdao Agricultural University, Qingdao 266109, China; Liangwang2019@qau.edu.cn

**Keywords:** chloride ion penetration, strength, mineral admixtures, petroleum coke residue, waste

## Abstract

Taking advantage of the desulfurization petroleum coke residue obtained from circulating fluidized bed boiler technology to replace a part of cement clinker and prepare the concrete can not only reduce the production of cement clinker and related CO_2_ emissions, but can also improve the utilization rate and utilization level of petroleum coke waste, which has good environmental and economic benefits. In this study, through the comprehensive analysis of a compressive strength test, X-ray diffraction test, and Cl^−^ penetration resistance test, the hydration mechanism of desulfurized petroleum coke residue in concrete is revealed, and the optimum replacement ratios of single-added petroleum coke residue, multi-added petroleum coke residue, and mineral admixtures in concrete are evaluated and proposed. The results showed that mixing the 10% petroleum coke residue and 40% blast furnace slag would be most appropriate to replace the cement in concrete, thus the effective utilization of mineral admixtures and coke residue in concrete without strength loss could be realized.

## 1. Introduction

**Figure 1:**
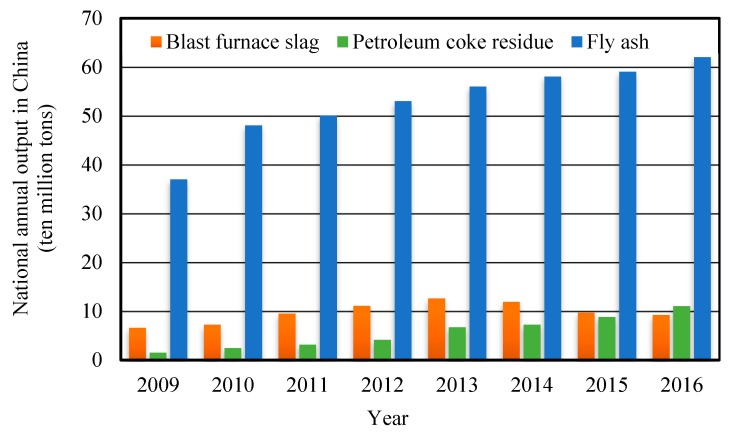
National annual output of industrial wastes. (Source: National Bureau of Statistics-Environmental Statistics Yearbook.)

For the oil refining industry, petroleum coke is the final product of petroleum after delayed coking treatment; the petroleum coke residue is the solid waste produced by the combustion and utilization of petroleum coke [1]. According to the data of “National Bureau of Statistics- Environmental Statistics Yearbook” [2], the national annual output of different solid industrial wastes in China is shown in Figure 1. The Sulphur content of petroleum coke is as high as 5–8%, which is not suitable for use as a raw material for chemical and metallurgical industries with high quality requirements [3]. Meanwhile, petroleum coke produces Sulphur dioxide after combustion as fuel, which seriously pollutes the atmosphere environment and affects human health. Therefore, it is very necessary to desulfurize high sulfur petroleum coke during combustion for utilization.

**Figure 2:**
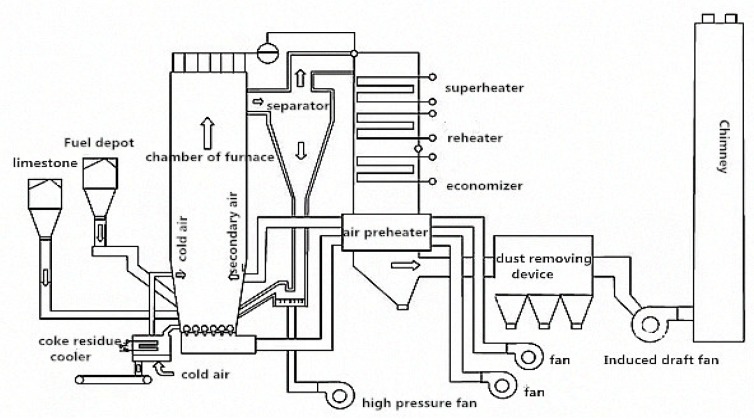
Schematic diagram of circulating fluidized bed combustion technology (CFB). (Source: ZHENGGUO VESSEL CO. LTD.).

Circulating fluidized bed combustion technology (CFB) is a rapidly developing clean combustion technology with high efficiency and low pollution in recent years [4,5]. The high sulfur petroleum coke mainly adopts lime powder desulfurization technology. The limestone is ground into powder to mix with the petroleum coke after refining, and then sprayed into the fluidized bed boiler for combustion. With the CaO from high temperature decomposition of limestone reacted with SO_2_ from the combustion of petroleum coke to form the CaSO_4_, the desulfurization efficiency can reach up to 90% [6]. The Schematic diagram of CFB technology is shown in Figure 2 (source: ZHENGGUO VESSEL CO. LTD., Zhengzhou, China) [7]. The desulfurization petroleum coke product can be divided into desulfurization petroleum coke ash and coke residue; the ash residue collected from dust removal system is called petroleum coke ash, while the waste residue remains in the bottom of the bed boiler is called petroleum coke residue [8]. So far, the comprehensive application of petroleum coke residue in general project constructions are still rare. Most are used for reclamation, while a small part is used as additives in cement plants [9]. With the wide application of CFB in China, the production of desulfurization petroleum coke residue will grow exponentially and is expected to be more than 150 million tons in 2019, which will bring great pressure to economic construction and the ecological environment, so the resource recycling of desulfurization petroleum coke residue needs to be solved urgently [10].

Desulfurization petroleum coke residue contains a large amount of lime and gypsum composition, which has a good cementitious property. Using the desulfurization petroleum coke residue to replace a part of cement clinker to prepare the concrete can not only reduce the production of cement clinker and related CO_2_ emissions, and protect the environment and land resources, but can also improve the utilization rate of petroleum coke residue, which has important significance in resource saving and waste recycling. Desulfurization petroleum coke residue can provide a lot of calcium materials and free Ca(OH)_2_, and the pozzolanic effect of mineral admixtures could be stimulated to achieve twice hydration and further improve the strength of concrete. Therefore, in this study, the optimum replacement ratios of single-added petroleum coke residue, multi-added with the petroleum coke residue with fly ash and blast furnace slag to replace the cement in the concrete, were both studied and discussed. The obtained results have great importance in using the desulfurization petroleum coke residue to replace the cement in general project construction.

## 2. Experimental Programs

### 2.1. Experimental Materials

**Figure 3:**
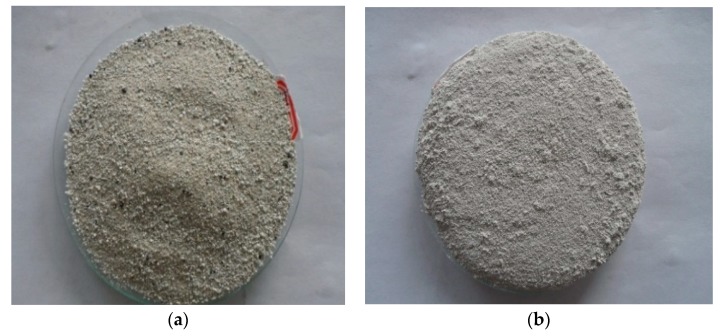
Petroleum coke residue in this study: (**a**) before grinding; (**b**) after grinding.

**Figure 4:**
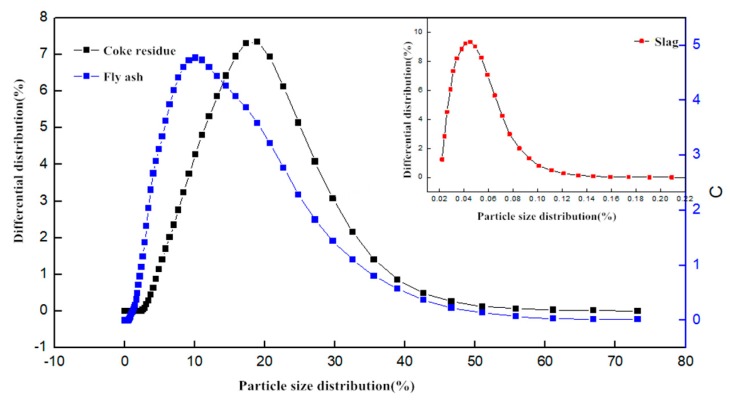
The particle size distribution of different mineral admixtures.

Ordinary Portland cement (PO 42.5R), which met the requirements of National Standard of China (GB/T) 1346-2011 [11], was used in this study. The detailed parameters of aggregates are shown in Table 1 and Table 2. Ordinary II-level fly ash (density is 2.24 g/cm^3^) and ground granulated blast furnace slag (density is 2.88 g/cm^3^) were used. The obtained petroleum coke residue from the refinery was vibrated and finely-ground by ball-mill (LAIZHOU WANKAI MACHINERY CO., LTD., Laizhou, China) to be taken as raw materials. The ball mill is a horizontal cylindrical rotating device driven by external gears. The centrifugal force produced by the rotation of the cylinder makes the steel ball roll continuously and grinds the petroleum coke residue. The photos of petroleum coke residue before grinding and after grinding are shown in Figure 3. The fineness of petroleum coke residue before grinding is similar to that of sand, and the particle size distribution of coke residue after grinding is similar to that of ordinary fly ash The specific surface area tested by BET Automatic Measuring Instrument (F-Sorb 2400-BET, Gold APP Instrument Corporation, Beijing, China) was 4065 cm^2^/g. The particle size distribution of coke residue, fly ash, and ground granulated slag is shown in Figure 4.

**Figure 5:**
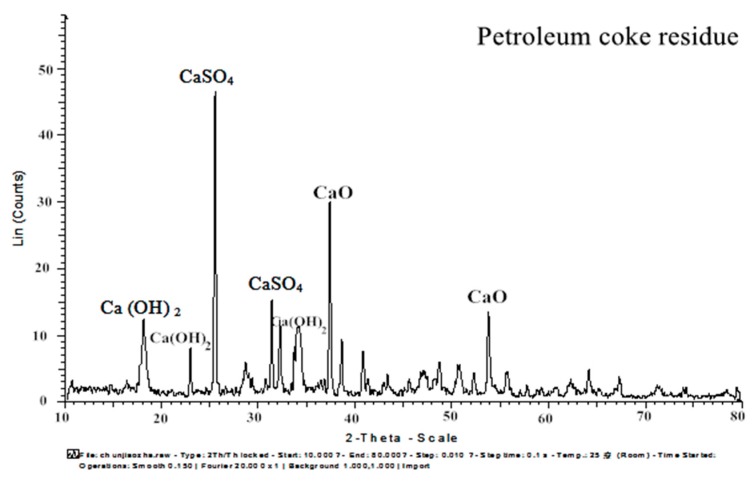
XRD map of petroleum coke residue.

X-ray diffraction (XRD, D8 Advance, Bruker AXS, Germany) and X-ray fluorescence (XRF, S8 Tiger, Bruker AXS, Germany) were performed to characterize the cement and mineral admixtures. The XRD map of petroleum coke residue is shown in Figure 5. The obvious diffraction peaks of CaSO_4_ II (2θ = 25.53, 31.46, 32.15) and CaO (2θ = 38.58, 53.84) can be found in the figure. In order to determine the proportion of CaSO_4_ II and CaO in desulfurized petroleum coke residue, an XRF test was carried out. Table 3 provides the chemical compositions of cement, fly ash, and blast furnace slag, as well as petroleum coke residue, as measured by XRF analysis. Because the XRF analysis will offer the content of carbon element inaccurate measurement, the loss of ignition test of petroleum coke residue was also conducted. Through the calculations, the CaSO_4_ II content is 39.98%, CaCO_3_ content is 3.18%, and free CaO content is 43.42%. In addition, it contained 3.7% amorphous carbon (C), 1.4% CO_2_, and other impurities. Therefore, it can be concluded that the sulfur content of petroleum coke residue mainly exists in the form of CaSO_4_ II, while the remaining calcium element mainly exists in the form of CaO.

### 2.2. Mix Proportions Design

The mix proportions of single-added petroleum coke residue concrete. Multi-added petroleum coke residue with mineral admixtures concrete are shown in Table 4 and Table 5, respectively. The mark “CR” represents petroleum coke residue, the mark “C” represents cement, the mark “BFS” represents blast furnace slag, and the mark “FA” represents fly ash. The marks “S”, “G”, and “Ad” represent the fine aggregate, coarse aggregate, and superplasticizer admixture, respectively. For single-added coke residue concrete, a water to binder ratio (W/(CR + C)) of 0.5 was used to prepare the specimens. The replacement ratios of petroleum coke residue were designed as 0, 10, 15, 20, and 30, respectively. Regarding the mix proportion of multi-added coke residue concrete, because the upper limit of fly ash’s effective replacement ratio in concrete is 40% based on the previous research [12], combining with 10% coke residue is likely to further improve the strength because of its twice hydration and micro-aggregate effect. The proportion of cement and total mineral admixtures in concrete was designed as 1:1. Because the maximum replacement ratio of II-level fly ash in concrete is regarded as 50% in China according to the national standard “Fly Ash Used in Concrete and Cement” [13], the blank group of single-added 50% fly ash was also carried out. Similarly, the replacement ratios of coke residue in the mineral admixtures were designed as 0, 10, 15, 20, and 30, respectively. The slump of different concrete was kept in the range of 180–220 mm by adjusting water consumption.

### 2.3. Experimental Methods

Based on “Standard for test method of mechanical properties of ordinary concrete” (GB/T50081-2002) [14] in China, three identical cubic concrete specimens were made into 100 mm × 100 mm × 100 mm for each specimen design. The curing of concrete specimens was carried out in a constant 20 ± 2 °C curing temperature and 50–60% humidity laboratory. The compressive strength tests of concrete were conducted at 3, 7, 14, and 28 days, respectively. At the same time, the X-ray diffraction (XRD) analysis test was performed to characterize the different concrete at 28 days by XRD device. Moreover, according to the national standard of GB/T50082-2009, the chloride ion penetration coefficient test for concrete was also measured through the Rapid Chloride Migration (RCM) Method [15]. By exerting an external electric field for concrete to accelerate the migration velocity of chloride ion in concrete, the penetration depth of chloride ion in concrete in a certain time can be tested. When combined with the Nernst-Plank equation, the measured numerical values, which are thickness of specimens, electric time, and other parameters, are put into the Equation (1), the penetration coefficient of Cl^−^ in concrete can be calculated out [16].

(1) $$\begin{array}{cc} D_{\mathrm{RCM}}= & 2.872\times 10^{-6}\frac{Th(x_{d}-\alpha \sqrt{x_{d}})}{t}\\ \alpha = & 3.338\times 10^{-3}\sqrt{Th} \end{array}$$

where $D_{\mathrm{RCM}}$—Cl^−^ penetration coefficient (m^2^/s); *T*—Average value of initial and final temperature (K); *h*—Height of specimens (m); $x_{d}$—Cl^−^ penetration depth (m); *t*—Electric testing time(s); *α*—Auxiliary variable.

**Figure 6:**
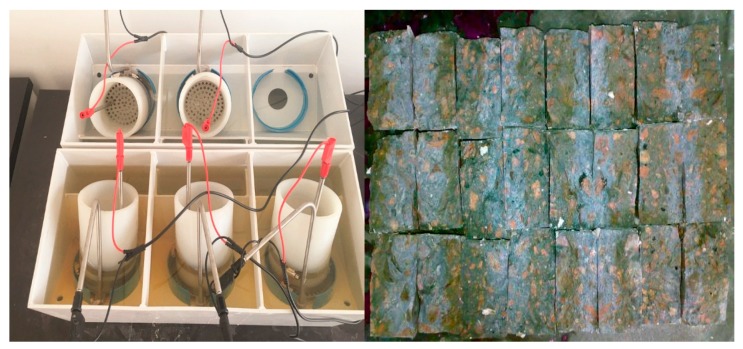
Cl^−^ penetration device and testing specimens.

The specimens of concrete were made into a cylinder with a diameter Φ (100 ± 1) mm and a height of h (50 ± 2) mm, and then were put in a water tank to start the water-curing in a standard curing room. At the age of testing, the specimens were submerged in an ultrasonic bath for 15 min before starting the test. After that both sides of the test specimens were linked with a 40 V voltage, then the positive and negative electrodes of specimens were immersed in the 0.2 mol/L KOH solution and 0.2 mol/L KOH solution containing 5%NaCl, respectively. The electric time was determined according to the initial electric current. After the electric current was over, the specimens were chopped into two halves by a pressure machine, and a 0.1 mol/L AgNO_3_ solution was sprayed on the surface of split specimens. The penetration coefficient of chloride ions of concrete was calculated by the penetration depth of chloride ions. The Cl^−^ penetration device and testing specimens are shown in Figure 6.

Considering that the CaO content of petroleum coke residue is high and easy to absorb moisture in the air and convert to Ca(OH)_2_, the petroleum coke residue was placed without coverage under the condition of 20 ± 2 and 50~60% humidity for a week in this study. According to JCT478.1-92 “Test Method of Building Lime-Physical Test Method” [17], the slacking velocities of newly-produced petroleum coke residue and old coke residue stored for a week and quick lime were determined. A total of 80 mL distilled water at 20 °C was added to the thermos flask, then the 40 g sample was poured into the thermos flask, and the stopwatch was started immediately. Timing began when the sample was poured into the water, recording the time when the maximum temperature was reached and when the temperature began to decrease. The time required to reach the maximum temperature was recorded as the digestion rate.

## 3. Results and Discussion

### 3.1. Exothermic Characteristic Curve of Petroleum Coke Residue

**Figure 7:**
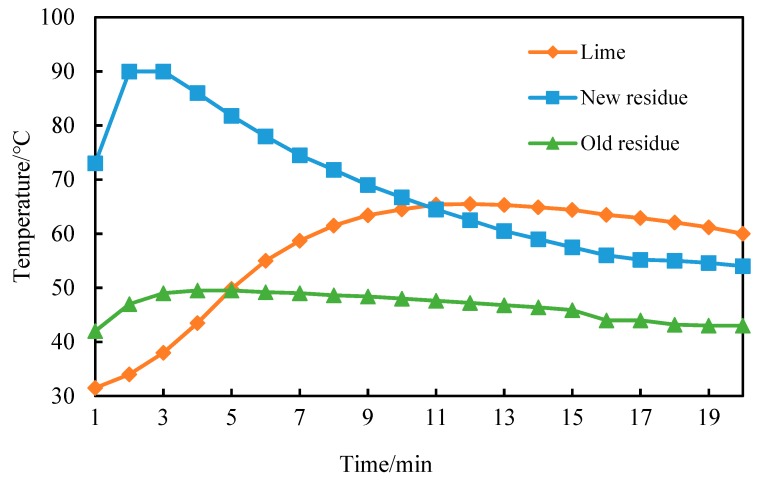
Exothermic characteristic curve of new residue, old residue, and lime.

From Figure 7, it can be seen that the CaO content of newly-produced coke residue is high, reacts rapidly with water, and due to fast heating speed, the heat release and slacking can basically be completed in a relatively short time (2 min). The highest temperature reached 90 °C, then the temperature decreased rapidly with heat dissipation. The decreasing ratio slowed after 10 min; the curve basically became gentle after 15 min and remained constant at about 60 °C. Because of the partial reaction of water in the air and old coke residue, the Ca(OH)_2_ and CaCO_3_ formed on the surface would block the reaction of water and internal CaO, leading to a slower heat release of old coke residue. The highest temperature reached 50 °C at 4 min, then the temperature remained constant at 45~49 °C. Compared with the petroleum coke residue, the slacking exothermic curve of lime increased slowly, and it reached the highest temperature at 12 min, but then the temperature remained above 60 °C.

### 3.2. Hydration Mechanism of Desulfurized Petroleum Coke Residue

According to XRF analysis and XRD analysis results of desulfurized petroleum coke residue, the desulfurized petroleum coke residue mainly consists of CaO and CaSO_4_. In order to study the hydration reaction mechanism and the stability of CaSO_4_ of desulfurized petroleum coke residue, in this study, samples of petroleum coke residue-cement paste were prepared and cured at room temperature of 20 °C. After 3 h, 6 h, 12 h, and 24 h, the samples were soaked in alcohol for 7 days. After being dried at 45 °C in a drying oven, X-ray diffraction tests were conducted to analyze the hydration of petroleum coke residue. 

**Figure 8:**
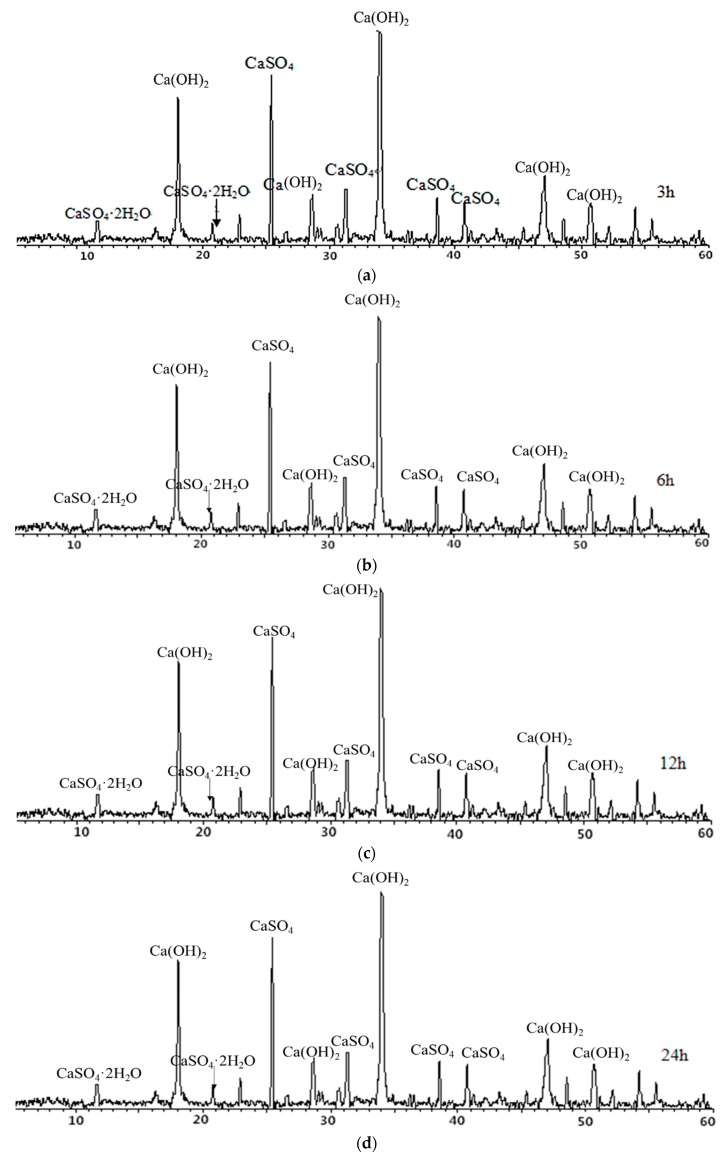
X-ray diffraction (XRD) maps of petroleum coke residue paste at (**a**) 3 h, (**b**) 6 h, (**c**) 12 h, and (**d**) 24 h.

From Figure 8, it can be seen that CaO in petroleum coke residue reacts with water to form Ca(OH)_2_, and the height change of the Ca(OH)_2_ diffraction peak is not obvious during the time range of 3 h–24 h, which indicates that CaO reacts with water rapidly. Moreover, there are weak diffraction peaks of CaSO_4_·2H_2_O at 2θ = 11.611, 20.758, which shows that CaSO_4_ can be partially hydrated to CaSO_4_·2H_2_O at 20 °C. However, the characteristic peaks of CaSO_4_ are still high in the above cases, which proves that hydration rate of CaSO_4_ is very slow and the degree of hydration reaction is insufficient at this time. This result of petroleum coke residue paste is similar to that of natural anhydrite at 20 °C curing condition, with a slow hydration process and low hydration rate [18,19].

**Figure 9:**
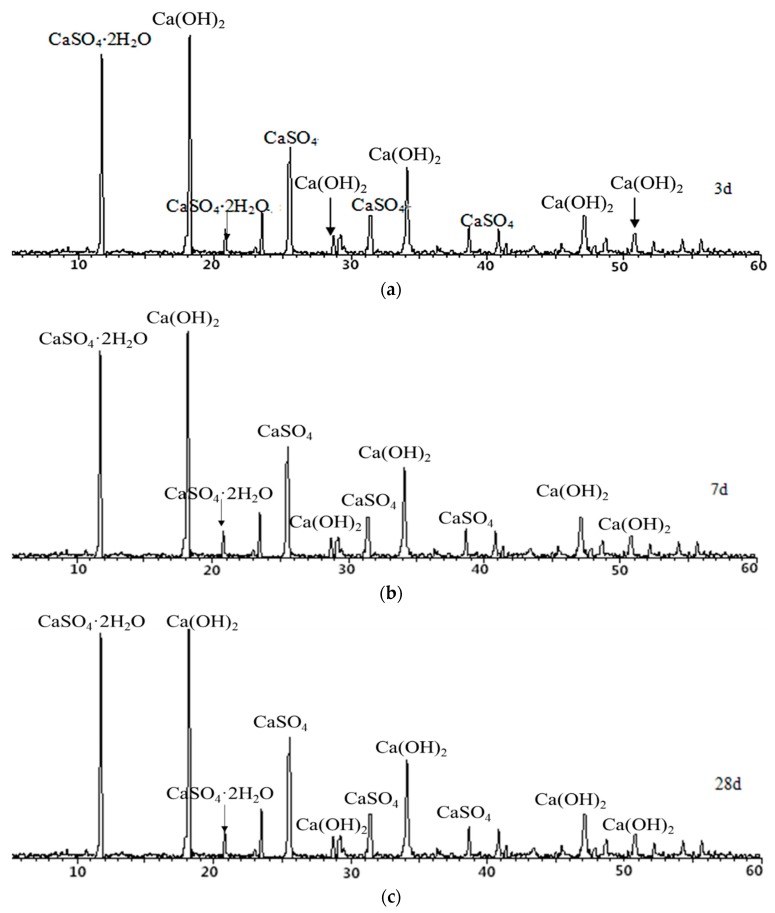
XRD maps of petroleum coke residue paste at (**a**) 3 days, (**b**) 7 days, and (**c**) 28 days.

In order to further study the hydration of desulfurized petroleum coke residue under room temperature and long curing time, a standard consistency paste of petroleum coke residue was prepared. The paste was cured for 3, 7, and 28 days under standard conditions, respectively, and then XRD analysis was carried out. The results are shown in Figure 9. From Figure 9, the characteristic peak of CaSO_4_ tends to weaken with the increase of curing age. The obvious characteristic peak of CaSO_4_·2H_2_O appears in the XRD map, which proves that under the standard curing condition, CaSO_4_ in petroleum coke residue is partially hydrated to CaSO_4_·2H_2_O. The peak value of CaSO_4_·2H_2_O in Figure 9 is higher than that in Figure 8, which indicates that hydration degree becomes more sufficient with the increase of curing time. However, according to the change of peak value of CaSO_4_·2H_2_O in Figure 9, the amount of CaSO_4_·2H_2_O is basically stable after 3 days. It might be that CaSO_4_·2H_2_O formed after hydration is wrapped on the surface of anhydrite minerals, which prevents further hydration of CaSO_4_. The hydration law of CaSO_4_ in anhydrite is similar to that of petroleum coke residue; the hydration reaction is basically stable after 3 days, and can be further improved in a short time by improving the fineness of anhydrite and adding different activators [20].

### 3.3. Optimum Replacement Ratio of Single-Added Petroleum Coke Residue in Concrete

#### 3.3.1. Compressive Strength Development

**Figure 10:**
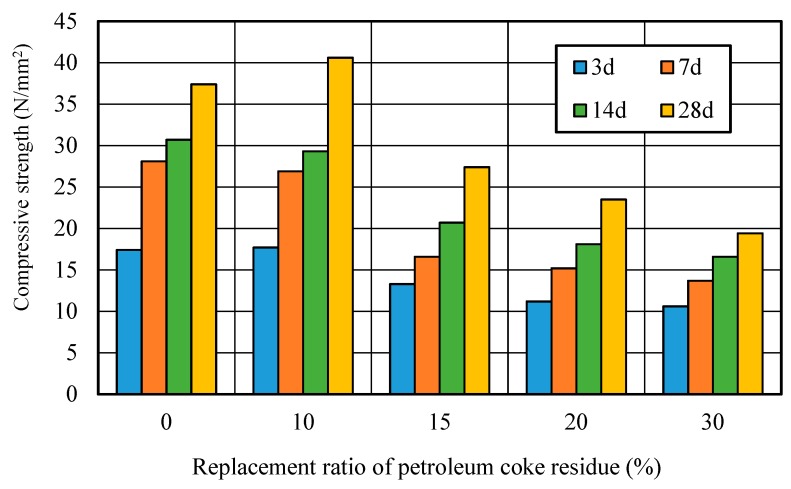
Strength of petroleum coke residue concrete with different replacement ratios.

Figure 10 showed the relationship between the compressive strength and replacement ratio of petroleum coke residue in concrete. It can be seen that the strengths first increased in the beginning, and then decreased along with the increase of replacement ratio. At 3 days, the strength of concrete at the replacement ratio of 10% reached the same level as that of ordinary concrete. Although the strength of concrete at the replacement ratio of 10% was slightly lower than that of ordinary concrete at 7 and 14 days, there was an obvious improvement in the strength at 28 days, where it increased by about 11% more than the ordinary concrete. However, when the replacement ratio was high and exceeded 10%, the strength of the petroleum coke residue concrete decreased sharply with the replacement ratio, decreasing by about 30%, much lower than that of ordinary concrete. Therefore, the replacement rate of 10% is reasonable for improving the strength of petroleum coke residue concrete.

#### 3.3.2. X-ray Diffraction Analysis (XRD)

**Figure 11:**
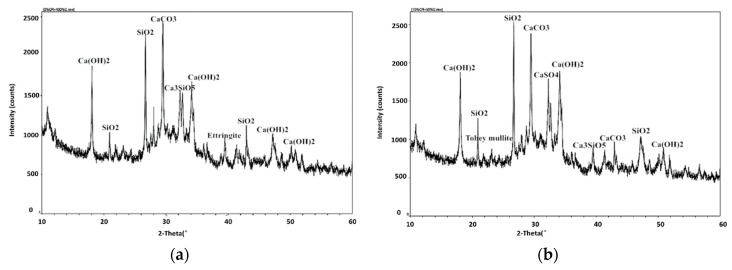
XRD maps of different concrete specimens at 28 days: (**a**) Ordinary cement; (**b**) 10% coke residue, and 90% cement.

Figure 11 shows the XRD analysis maps of petroleum coke residue concrete. It can be seen that due to the rapid hydration exothermic, the hydration reaction of coke residue in concrete was complete at 28 days. For the map of ordinary concrete, as shown in Figure 11a, the diffraction peaks of CaCO_3_ (2θ = 28.518) and SiO_2_ (2θ = 26.326) were relatively strong as major peaks, followed by the hydration product—Ca(OH)_2_. The zigzag curve around Ca(OH)_2_ diffraction peaks can be considered to be the precursor of calcium silicate hydrate gel. Meanwhile, the diffraction peaks of Ca_3_SiO_5_ (C_3_S) were also found. From Figure 11b, the replacement ratio of coke residue was 10%. Apart from the diffraction peaks of CaCO_3_ and SiO_2_ being relatively strong, the diffraction peaks of CaSO_4_ II (2θ = 19.401, 33.020, 36.408), Tobey mullite (C_5_S_6_H_5_, 2θ = 7.778, 23.170, 31.128), and ettringite (AFt) were also found. 

Based on the above results, when the replacement ratio of petroleum coke residue is 10%, on the one hand, the content of CaSO_4_ II in coke residue was relatively high, and the effect of CaSO_4_ II was similar to gypsum and played a role in retarding, which can produce a lot of hydration heat and shorten the setting time. High hydration heat would take away a part of the moisture, leading to a decrease of actual W/C ratio. At the same time, it can inhibit the stomatal irregular and uneven distribution caused by the hydration of cement at the early stage; on the other hand, the SO_3_ in coke residue can boost the reaction between SiO_2_, Al_2_O_3_, and hydration product to produce the Torbay mullite, which can be filled between particles and can improve the strength. Moreover, the CaSO_4_ II in coke residue could react with hydrated calcium aluminate to produce the ettringite (AFt), because the ettringite is insoluble in water and precipitated in the cement particles inside, leading to a strength improvement of concrete. However, the strength of concrete decreases when the replacement ratio of petroleum coke residue exceeds 10%. This is because with the increase of replacement ratio of coke residue, the ratio of calcium to silicon in the paste increases gradually, and it is very easy to form calcium silicate hydrate with lower strength, thus reducing the strength. It is also possible that excessive gypsum will lead to volume expansion and destruction of concrete. These results and reasons are similar to those of petroleum coke ash concrete, and the optimum replacement rate of petroleum coke ash in concrete is 15% [21].

#### 3.3.3. Relationship of SiO_2_/(C_2_S + C_3_S), CaSO_4_/(CaO + MgO + Al_2_O_3_) Ratio and Strength

**Figure 12:**
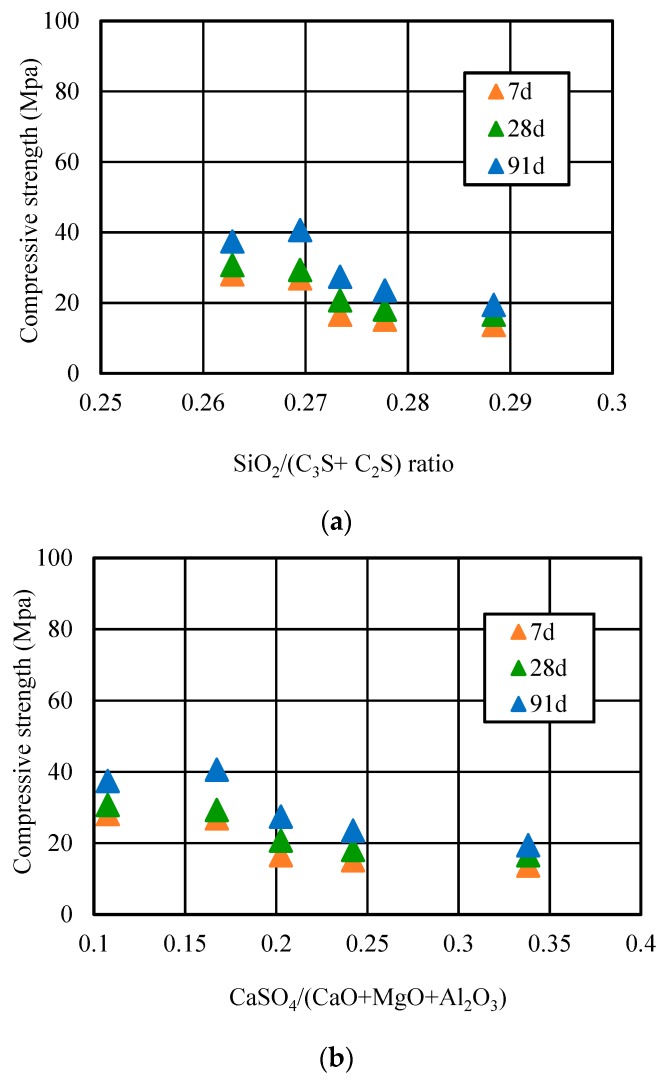
Relationship of (**a**) SiO_2_/(C_2_S + C_3_S) and (**b**) CaSO_4_/(CaO + MgO + Al_2_O_3_) ratio and strength.

Figure 12 showed the relationship between the SiO_2_/(C_2_S + C_3_S) ratio and compressive strength, and the CaSO_4_/(CaO + MgO + Al_2_O_3_) ratio and compressive strength of coke residue concrete. According to the Bogue Equations (2) and (3) [22], taking advantage of the parameter data in Table 3, the C_2_S and C_3_S amount of concrete with different replacement ratios of coke residue at different curing ages can be calculated finally.

C_3_S = 4.07 × CaO − (7.60 × SiO_2_ + 6.72 × Al_2_O_3_ + 1.43 × Fe_2_O_3_ + 2.85 × SO_3_) (2)

C_2_S = 2.87 × SiO_2_ − 0.754 × C_3_S (3)

It can be seen that when the replacement ratio was 10%, the SiO_2_/(C_2_S + C_3_S) ratio of concrete was 0.27, and the compressive strength reached the maximum at 91 days. However, when the replacement ratio was too high and more than 10%, along with the increasing replacement ratio, the SiO_2_/(C_2_S + C_3_S) ratio decreased gradually and the calcium silicon (Ca/Si) ratio gradually increased. This means it would be very easy to form a low strength hydrated calcium silicate, which also can lead to a decrease of strength. Moreover, the relationship curve of the CaSO_4_/(CaO + MgO + Al_2_O_3_) ratio and the compressive strength of petroleum coke residue concrete first increased, and then decreased when the replacement ratio exceeded 10%. The CaSO_4_/(CaO + MgO + Al_2_O_3_) ratio in concrete was over 0.17 and too high; the high CaSO_4_ amount in concrete would accelerate the setting of cement, resulting in a false setting phenomenon, which has a bad effect on the strength of concrete. In addition, too much SO_3_ of petroleum coke residue would lead to more formation of ettringite (Aft). Along with prolonged hydration time, the ettringite and high CaSO_4_ amount would be likely to become an unstable factor and could easily cause the volume expansion of concrete [23].

#### 3.3.4. Chloride Ion Penetration Property (RCM Method)

**Figure 13:**
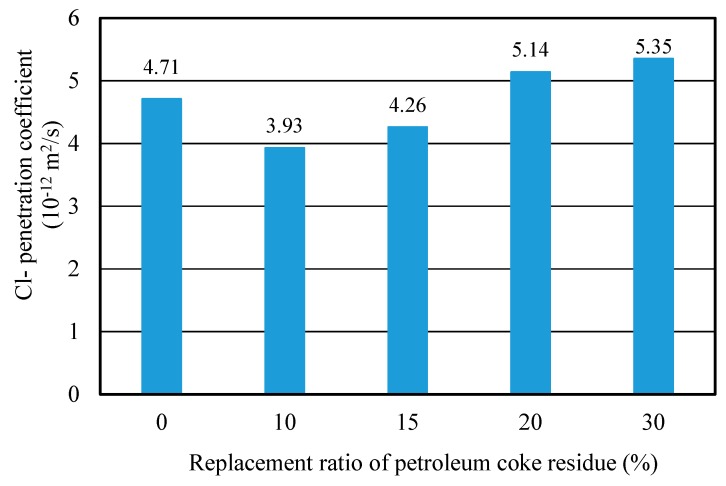
Cl^−^ penetration coefficient of coke residue concrete with different replacement ratios.

Figure 13 showed the Cl^−^ penetration coefficients of concrete with different replacement ratios of coke residue at 28 days. It can be observed that the curve of Cl^−^ penetration coefficient first decreased and then increased. When the replacement ratio of coke residue was 10%, the Cl^−^ penetration coefficient was the lowest and the resistance-penetration performance was the best; when the replacement ratio of coke residue was more than 10%, the Cl^−^ penetration coefficient of concrete increased with the replacement ratio. This revealed that 10% petroleum coke residue in concrete has a fine filling and micro-aggregate effect. It can produce a more solid C-S-H gel and small size hydration products to fill the pores, improving the pore structure and compactness of the concrete. The internal penetration of chlorine ions in concrete probably could be hindered [24]. This result was in agreement with compressive strength. In addition, it is noteworthy that although the Cl^−^ penetration coefficient of concrete at 15% was also lower than that of ordinary concrete, the CaSO_4_ amount of concrete was relatively high and the strength of concrete was lower than ordinary concrete. Therefore, from the results discussed above, it can be determined that the upper limit of effective replacement ratio of single-added petroleum coke residue in concrete was 10%. With a better strength improvement, the strength of concrete will decrease obviously when the replacement ratio of petroleum coke residue exceeds 10%.

### 3.4. The optimum Proportion of Multi-Added Petroleum Coke Residue with Mineral Admixture in Concrete

#### 3.4.1. Compressive Strength Development

**Figure 14:**
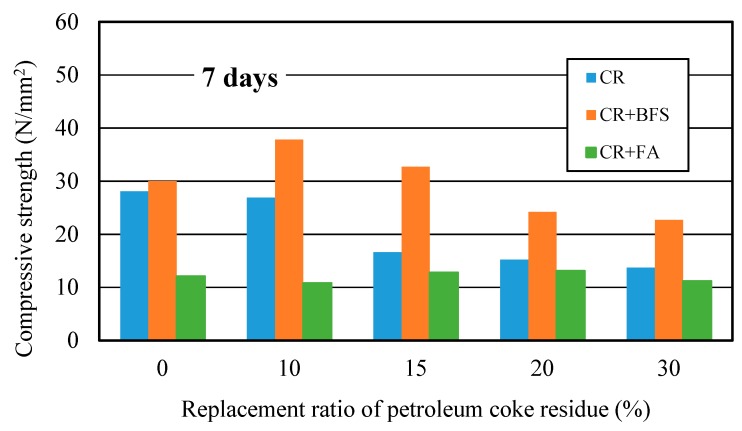

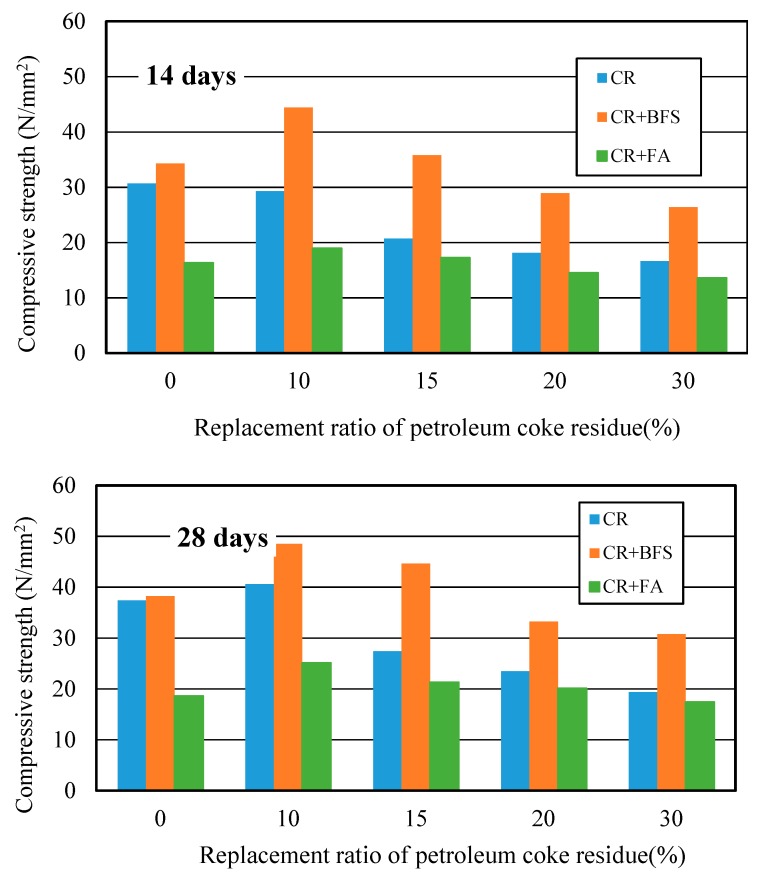
Compressive strengths of multi-added coke residue and mineral admixture concrete.

Figure 14 showed the strength development of three kinds of concrete at different curing days. It can be observed that the strengths of (CR+BFS) concrete at each curing age were all the highest, the strength improvement effect was more significant than single-added CR concrete, while the strength improvement effect of (CR + FA) concrete was the worst. Compared with ordinary concrete, the strength of single-added CR concrete at the replacement ratio of 10% increased by 11% at 28 days; for the (CR + BFS) concrete, when the replacement ratio of coke residue was 0% (50% cement and 50% slag), the strength of concrete reached the same level as ordinary concrete at each age; the strength of multi-added (CR + BFS) concrete at the replacement ratio of coke residue of 10 and 15% at 7 days increased by about 30.4% and 11% more than that of ordinary concrete, respectively. At 28 days, when the replacement ratio of coke residue was 10 and 15%, the strengths of (CR + BFS) concrete increased by 32% and 19%, respectively. In contrast, the strength of (CR + FA) concrete at each replacement ratio was the lowest, due to the slow hydration of fly ash and the high water absorption ratio of petroleum coke residue in concrete. Most of the water was absorbed by coke residue, which is not conducive to the activation of the pozzolanic effect of fly ash, causing that the strength of (CR + FA) concrete to be very low. Therefore, it can be proved that the multi-added coke residue and blast furnace slag is more advantageous for enhancing the strength of concrete, and the strength improvement effect of 10% coke residue and 40% slag to replace the cement in concrete was the best.

#### 3.4.2. X-ray Diffraction Analysis (XRD)

**Figure 15:**
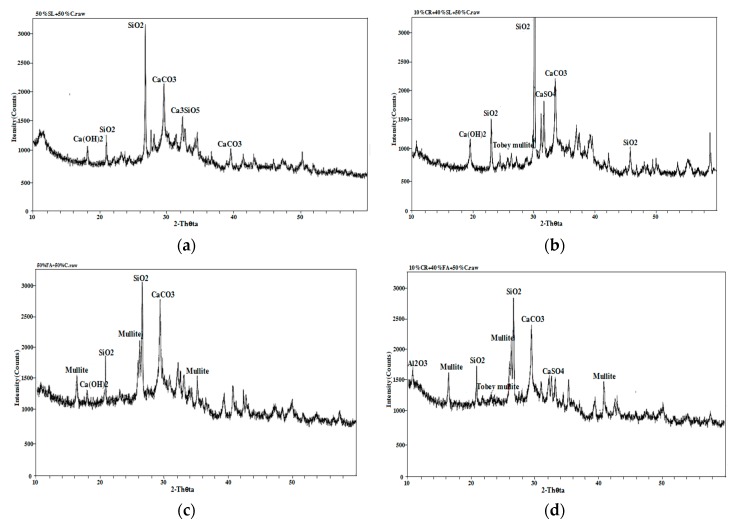
XRD maps of different concrete specimens: (**a**) 50% slag and 50% cement; (**b**) 10% coke residue, 40% slag, and 50% cement; (**c**) 50% fly ash and 50% cement; (**d**) 10% coke residue, 40% fly ash, and 50% cement.

From Figure 15, it can be seen that compared with the ordinary concrete, the SiO_2_ diffraction peak of 50% Blast furnace slag (BFS) and 50% cement concrete in Figure 15a became much stronger and the diffraction peaks of Ca(OH)_2_ and CaCO_3_ were obviously reduced. A large amount of SiO_2_ content in multi-added concrete made up for the strength loss caused by the reduction of cement hydration products, coupled with the micro-aggregate effect of slag in concrete, leading to the same strength level as ordinary cement concrete at 28 days. From Figure 15b, it can be seen that the SiO_2_ diffraction peaks of 10% petroleum coke residue (CR) and 40% BFS concrete became much stronger, the diffraction peak of Ca(OH)_2_ increased obviously over that of 50% BFS and 50% cement concrete, the hydration reaction was promoted further, and the diffraction peaks of CaSO_4_ and Tobey mullite were found, which could enhance the strength of concrete. From Figure 15c, compared with ordinary concrete, the SiO_2_ diffraction peaks of 50% fly ash and 50% cement concrete changed little, the diffraction peaks of CaCO_3_ and Ca(OH)_2_ decreased, and the diffraction peak of the hydration product was very small. Meanwhile, large diffraction peaks of were found for mullite, which came from artificial heating of aluminum silicate during the process of high temperature calcination [25]. Although the XRD map (Figure 15d) of 10% coke residue and 40% fly ash concrete also found the diffraction peaks of CaSO_4_ and Tobey mullite, many diffraction peaks of mullite were found, which may be the main reason why the strength of fly ash-coke residue concrete was much lower than that of ordinary concrete [26].

#### 3.4.3. Chloride Ion Penetration Property (RCM Method)

**Figure 16:**
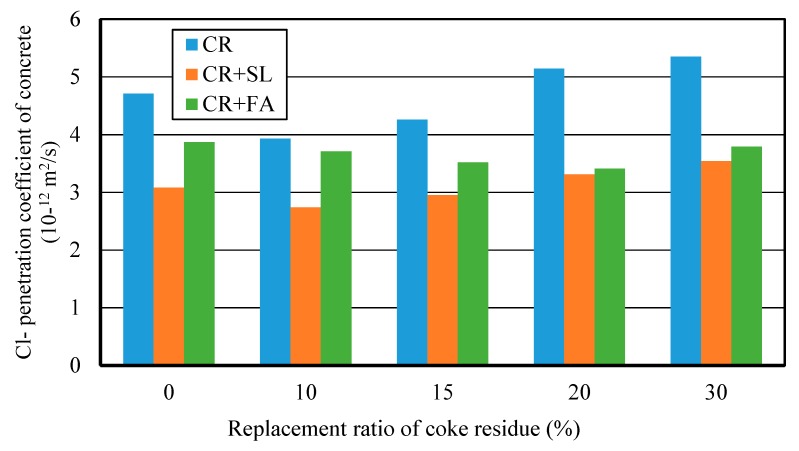
Cl^−^ penetration coefficients of different kinds of concrete at 28 days.

Figure 16 showed the relationship between Cl^−^ penetration coefficient and replacement ratio of petroleum coke residue for three kinds of concrete. It can be seen that the addition of mineral admixture can further improve the internal pore structure and compactness of petroleum coke residue concrete. The Cl^−^ penetration coefficient of (CR + BFS) concrete was the lowest among the three kinds of concrete, the Cl^−^ penetration coefficient of single-added CR concrete was the highest, and (CR+FA) concrete was in middle. This showed that mixing the mineral admixture and petroleum coke residue has a better grading and filling effect, and while it is likely to produce the more solid C-S-H gel and hydration products to fill the pores and the gap between coarse aggregates, the internal penetration of chlorine ions can be hindered [27]. In particular, for the multi-added (CR + BFS) concrete, when the replacement ratios of petroleum coke residue and BFS were 10% and 40%, the Cl^−^ penetration coefficient reached the lowest value. When the replacement ratio of coke residue in the concrete was too high, it was very easy for it to cause the volume expansion of concrete, which reduces the compaction degree of the internal structure of concrete. Combined with the results of strength, accordingly, the proposal here is that mixing 10% petroleum coke residue and 40% blast furnace slag would be most appropriate to replace the cement in the concrete, as this will achieve an optimum utilization of mineral admixtures and coke residue in concrete without strength loss. Approximately 50% cement content could be reduced, which would have good environmental and economic benefits. This is greatly important for effective utilization and maximization of different mineral admixtures in the general project construction industry.

In addition, the analysis of the properties before of many mineral admixtures before utilization as supplementary cementitious materials is often insufficient, which may lead to unexpected results in some cases. Through a system of physical and chemical techniques for the characterization of technogenic pozzolans in the future, the property analysis of coke residue concrete could be perfected [28].

## 4. Conclusion

In this study, through a comprehensive analysis, the following results were obtained in this study:

(1) In the case of single-added petroleum coke residue concrete, the strength of concrete reached the maximum value when the replacement ratio of coke residue was 10%, and then decreased sharply along with the increase of replacement ratio.
(2) The CaSO_4_Ⅱ in coke residue was similar to gypsum and played a retarding role, which can produce a lot of hydration heat and shorten the setting time. High hydration heat takes away moisture, leading to a decrease of actual water/cement ratio (W/C); on the other hand, the SO_3_ in coke residue can boost the reaction between SiO_2_, Al_2_O_3_, and the cement hydration product to produce the Torbay mullite, which can be filled between particles and improves the strength.
(3) Single-added 10% petroleum coke residue in concrete has a fine micro-aggregate effect. It is likely to produce a more solid C-S-H gel and small size hydration products to fill the pores, so as to improve the pore structure and compactness of concrete, which probably reduces the porosity of concrete. The internal penetration of chlorine ions was hindered, meaning the Cl^−^ penetration resistance property was improved.
(4) In the case of multi-added petroleum coke residue with mineral admixture concrete, through the comparative analysis, it can be seen that the way multi-added coke residue with blast furnace slag is more advantageous in enhancing the performance of concrete. The strength improvement and Cl^−^ penetration resistance effect of 10% coke residue and 40% slag to replace the cement in concrete was the best. At 28 days, when the replacement ratios of coke residue and slag were 10% and 40%, the strength increased by about 32% more than ordinary concrete.
(5) In contrast, the strength improvement effect of (CR + FA) concrete was the worst. Due to the slow hydration of fly ash and high water absorption rate of petroleum coke residue, most of the water was absorbed by the coke residue, which is not conducive to the activation of the pozzolanic effect of fly ash, together with the low strength mullite in fly ash, causing the strength of (CR + FA) concrete to be very low.
(6) Therefore, accordingly the proposal here, mixing 10% petroleum coke residue and 40% blast furnace slag would be most appropriate to replace the cement in the concrete, as this will achieve an optimum utilization of mineral admixtures and petroleum coke residue in concrete without strength loss. Approximately 50% cement content and related CO_2_ emissions could be reduced, which would have great environmental and economic benefits.

## Figures and Tables

**Table 1:** Physical results and particle size distribution of fine aggregate (natural river sand).

Apparent Density (g/cm^3^)	Bulk Density (g/cm^3^)	Water Absorption (%)	Sieve Size	4.75	2.36	1.18	0.6	0.3	0.15	Fineness Modulus
2.59	1.45	1.65	Accumulated retained percentage (%)	10	25	37	54	83	97	2.72

**Table 2:** Physical results and particle size distribution of coarse aggregate (granite stone).

**Water Absorption (%)**	1.70					
**Crushing Index (%)**	11.2					
**Bulk Density (kg/m^3^)**	1460					
**Apparent Density (kg/m^3^)**	2510					
**Needle and Plate Particle Content (%)**	4.05					
**Continuous Grading of Aggregate (mm)**	5–25					
**Sieve Size**	25	20	16	10	5	2.5
**Accumulated retained percentage (%)**	0.9	18.9	48.4	95.9	99	99.5

**Table 3:** Chemical composition results of different mineral admixtures by XRF.

Type	CaO	SO_3_	SiO_2_	Al_2_O_3_	MgO	Fe_2_O_3_	Others
Portland Cement	61.71	1.99	20.07	5.09	1.58	2.93	6.63
II-level Fly ash	2.41	0.35	57.36	28.68	1.28	4.29	5.63
Blast furnace slag	43.51	-	34.11	14.56	5.54	0.28	2
Petroleum coke residue	61.66	23.52	4.43	2.26	1.79	0.73	5.61

**Table 4:** Mix proportions of different concrete single-added petroleum coke residue.

No.	W/(C + CR) (%)	Replacement Ratio of CR (%)	C (%)	Unit Content (kg/m^3^)					
				W	C	CR	S	G	Ad.
CRC-1	50	0	100	177	350	0	762	1143	4.5
CRC-2		10	90	177	315	35	762	1143	4.5
CRC-3		15	85	177	298	52	762	1143	4.5
CRC-4		20	80	177	280	70	762	1143	4.5
CRC-5		30	70	177	245	105	762	1143	4.5

**Table 5:** Mix proportion of concrete multi-added petroleum coke residue and (slag/fly ash).

W/B (%)	CR + BFS(FA)/B (%)	CR (%)	BFS (FA) (%)	Unit Content (kg/m^3^)						
				W	C	CR	BFS (FA)	S	G	Ad.
50	50	0	50	177	175	0	175	762	1143	4.5
		10	40	177	175	35	140	762	1143	4.5
		15	35	177	175	52	123	762	1143	4.5
		20	30	177	175	70	105	762	1143	4.5
		30	20	177	175	105	70	762	1143	4.5

B = C + CR + BFS (FA).

## References

[1] Zheng B., Sun P., Liu Y.Q., Zhao Q. (2018). Heat transfer of calcined petroleum coke and heat exchange tube for calcined petroleum coke waste heat recovery. Energy.

[2] Bureau of Statistics-Environmental Statistics Yearbook: National Statistical Yearbook. http://data.gzstats.gov.cn/gzStat1/chaxun/njsj.jsp.

[3] Tripathi N., Singh R.S., Hills C.D. (2019). Microbial removal of sulphur from petroleum coke (petcoke). Fuel.

[4] Kang Y.K., Choi Y.C. (2018). Development of non-sintered zero-OPC binders using circulating fluidized bed combustion ash. Constr. Build. Mater..

[5] Tomasz K., Anna K., Ryszard C. (2019). Effective adsorption of lead ions using fly ash obtained in the novel circulating fluidized bed combustion technology. Microchem. J..

[6] Zhang X., Li Q.Y., Zhu S.H. (2009). Experimental Research on Mortar Made by Desulfurization Ash and Dregs. J. Qingdao Technol. Univ..

[7] ZHENGGUO Vessel Co. Ltd. Home Page: Circulating Fluidized Bed Boiler. https://www.zgrongqi.com/guolu/lhcguolu.html.

[8] Chen X., Li Q.Y., Wang F.C., Wang L. (2013). Basic Performance and Its Application of Desulfurization Petroleum Coke Residue. J. Qingdao Technol. Univ..

[9] Du H., Li Q.Y., Zhu S.H. (2010). Experimental study on aerated concrete with petroleum coke desulfurization slag. New Build. Mater..

[10] Shan Y.L., Guan D.B., Meng J., Liu Z., Schroeder H., Liu J.H., Mi Z.F. (2018). Rapid growth of petroleum coke consumption and its related emissions in China. Appl. Energy.

[11] (2011). Test Methods for Water Requirement of Normal Consistency, Setting Time and Soundness of the Portland Cement. National Standard of the People’s Republic of China (GB/T 1346-2011).

[12] Wang L., Uji K., Ueno A., Ohno K. (2015). Experimental Study on Upper Limit of Fly ash’s Effective Replacement Ratio in Mortar. Proceeding of 70th JSCE Annual Meeting in Okayama.

[13] (2005). Fly Ash Used in Concrete and Cement. National Standard of The People’s Republic of China (GB/T 1596-2005).

[14] (2003). Standard for test method of mechanical properties of ordinary concrete. National Standard of the People’s Republic of China (GB/T 50081-2002).

[15] (2010). Standard for test method of long-term performance and durability of ordinary concrete: RCM method. National Standard of the People’s Republic of China (GB/T 50082-2009).

[16] Dehghan A., Peterson K., Riehm G., Bromerchenkel L.H. (2017). Application of X-ray microfluorescence for the determination of chloride diffusion coefficients in concrete chloride penetration experiments. Constr. Build. Mater..

[17] (2013). Standard test method for building lime-Part 1: Methods for physical testing. National Standard of the People’s Republic of China (JCT478.1-2013).

[18] Jiang H., Li Q.Y., Yue G.B., Yu C.Z. (2014). The research on durability of petroleum coke desulfuration residues aerated concrete. New Build. Mater..

[19] Li D., Pan Z.H., Liu M.L., Zhou H.L., Han J.X., Zheng C.H. (2010). Study on hydration of natural anhydrite. J. China Non-Met. Min. Ind. Herald.

[20] Bai L., Peng J.H., Zhang J.X., Wan T.Z. (2008). Study on hydration and hardening of natural anhydrite. J. Non-Met. Mineral..

[21] Yu C.Z., Li Q.Y., Yue G.B., Jiang H. (2014). Desulfurization of petroleum coke residue ash ’experimental study on the mechanical behaviour of concrete. Concrete.

[22] Ogawa Y.K., Uji K., Ueno A. (2010). Evaluation of fly ash as cementitious material for strength. J. Cem. Sci. Concr. Technol..

[23] Yang J.S. (2003). Discussion on the action duality of ettringite and it’s causing condition in concrete. J. China Civ. Eng..

[24] Wang Y.L., Jin Z.Q., Liu S.X., Yang L., Luo S.Q. (2013). Physical filling effect of aggregate micro fines in cement concrete. Constr. Build. Mater..

[25] Han G.H., Yang S.Z., Peng W.J., Huang Y.F., Wu H.Y., Chai W.C., Liu J.T. (2018). Enhanced recycling and utilization of mullite from coal fly ash with a flotation and metallurgy process. J. Clean. Prod..

[26] Li C.X., Zhou Y., Tian Y.M., Zhao Y.Y., Wang K.Y., Li G.M., Chai Y.S. (2019). Preparation and characterization of mullite whisker reinforced ceramics made from coal fly ash. J. Ceram. Int..

[27] Yang T., Zhu H., Zhang Z.H., Gao X., Zhan C.S., Wu Q.S. (2018). Effect of fly ash microsphere on the rheology and microstructure of alkali-activated fly ash/slag pastes. Cement Concr. Res..

[28] Záleská M., Pavlíková M., Pavlík Z., Jankovský O., Pokorný J., Tydlitát V., Svora P., Černý R. (2018). Physical and chemical characterization of technogenic pozzolans for the application in blended cements. Constr. Build. Mater..

